# Chemical Composition and In Vitro Bioactivities of Extracts from Cones of *P. halepensis*, *P. brutia*, and *P. pinea*: Insights into Pharmaceutical and Cosmetic Potential

**DOI:** 10.3390/plants13131802

**Published:** 2024-06-29

**Authors:** Amel Chammam, Luc Fillaudeau, Mehrez Romdhane, Jalloul Bouajila

**Affiliations:** 1 Toulouse Biotechnology Institute, Bio & Chemical Engineering TBI (CNRS UMR5504, INRAE UMR792, INSA Toulouse), 31400 Toulouse, France; chammam@insa-toulouse.fr (A.C.); luc.fillaudeau@insa-toulouse.fr (L.F.); 2 Energy, Water, Environment and Process Laboratory (LR18ES35), National Engineering School of Gabes, University of Gabes, Gabes 6029, Tunisia; mehrez.romdhane@univgb.tn; 3 Laboratoire de Génie Chimique, Université de Toulouse, CNRS, INP, UPS, 31062 Toulouse, France

**Keywords:** *Pinus*, antioxidants, anticancer, polyphenols, HPLC, GC-MS

## Abstract

Various parts of the *Pinaceae* species, a traditional plant, have potential health benefits and exhibit antibacterial, anti-cancer, and antioxidant activities. This study aims to investigate the biochemical properties of both petal (P) and core (C) fractions from pinecones of *P. halepensis* (PA), *P. brutia* (PB), and *P. pinea* (PP). Pinecones were manually separated into P and C, which were then milled to investigate maceration with solvents of increasing polarity: cyclohexane (1SV), ethyl acetate (2SV), and methanol (3SV) at 20 °C. Spectrophotometry was utilized to quantify the total phenolic content (TPC) and to assess bioactivities. Gas chromatography with mass spectrometry (GC-MS) and high-performance liquid chromatography (HPLC) were employed to identify the chemical composition. 3SV extracts demonstrated the highest TPC and a significant anti–oxidant potential. PA-P-3SV exhibited the highest TPC (460.66 mg GAE/g DW) and PP-P-3SV displayed the best IC_50_ (10.54 µg/mL) against DPPH. 1SV and 2SV extracts showed interesting anticancer activity against Hela and HepG2 cells. No significant toxic effect of P and C extracts from pinecones was observed on HEK-293 cells. GC-MS analysis unveiled 46 volatile compounds, of which 32 were detected for the first time in these species. HPLC analysis identified 38 compounds, of which 27 were not previously detected in these species. This study highlights the significant potential of pinecones as a rich source of bioactive compounds.

## 1. Introduction

Forests are recognized as the main reservoir of medicinal plants and a diverse array of forest products, including fodder, fiber, food items, cosmetics, gums, perfumes, resins, dyes, and plant protectants [1,2]. Medicinal and aromatic plants within agroforestry systems play a crucial role in drug discovery for both animal and human healthcare. Particularly in villages and remote areas, where dependence on these plants and their derivatives is prevalent, they are often the primary choice for treating human ailments [3]. Worldwide, the search for plant products used in cosmetics is continuous. A recent trend in Western cosmetics involves incorporating ingredients from traditional Chinese medicine or Ayurvedic medicine, including extracts or compounds derived from plants, fungi, or animals [4]. Schmidt [4] presented various medicinal plant extracts widely used as active ingredients in cosmetics, including species from the *Pinaceae* family, the *Polygonaceae* family, and the *Rosaceae* family.

In recent years, extensive research has been dedicated to exploring the potential health advantages of antioxidants. Numerous synthetic and natural compounds have been thoroughly examined to evaluate their antioxidant properties [5]. Phenolic compounds are molecules with antioxidant properties that play roles in preventing heart disease [6,7], reducing inflammation [8], decreasing the occurrence of cancers [9,10] and diabetes [11], and lowering rates of mutagenesis in human cells [12].

The extraction of bioactive compounds from plants can be achieved by various methods. Over the past 50 years, non-conventional methods have been developed to be more environmentally friendly by reducing synthetic and organic chemicals, decreasing operational times, and improving the yield and quality of extracts [13]. Among these methods, Supercritical Fluid Extraction (SFE), Ultrasound-Assisted Extraction (UAE), and Microwave-Assisted Extraction (MAE) stand out. UAE uses high-intensity ultrasonic waves to disrupt cell walls, facilitating solvent diffusion. MAE uses electric and magnetic fields to enhance heat transfer and conduction, creating a dipole moment between the solvent and the sample [14]. The primary advantage of both UAE and MAE is the significant reduction in extraction time, consequently reducing solvent consumption and costs. However, these methods have limitations, including low selectivity, solvent usage, and energy consumption [14]. SFE employs supercritical fluids as solvents to extract bioactive compounds, offering several advantages due to the high diffusion coefficient and low viscosity of supercritical fluids, which allow them to penetrate the solid matrix pores easily, enhancing extraction efficiency [15]. The fluid density of a supercritical fluid is highly sensitive to changes in temperature and pressure at the critical point, enabling selective extraction by adjusting these conditions. Nevertheless, several drawbacks restrict the use of SFE, such as expensive setup and insufficient technological understanding of SFE characteristics [16]. Despite the advancements in non-conventional methods, traditional extraction methods such as Soxhlet extraction and maceration remain relevant. Soxhlet extraction has a dual role as a standard extraction method and a reference point for comparing new alternatives [17]. However, maceration stands out for its simplicity, minimal equipment requirements, low cost, and environmental friendliness. Maceration operates at lower temperatures and yields higher polyphenol content compared to other methods. The speed and duration of agitation are crucial factors in maceration, as they influence mass transfer rates and overall extraction efficiency [18]. Mahindrakar and Rathod [19] reported that maceration of *Syzygium cumini* seeds at 50 °C for 2 h yielded a higher total polyphenol content (TPC) (79.87 mg GAE/g) compared to that obtained via Soxhlet extraction at 100 °C for 6 h (30.05 mg GAE/g). Moreover, Shirsath et al. [20] reported a higher yield of curcumin at lower temperatures (30 °C) with maceration compared to Soxhlet extraction. These findings highlight maceration’s efficiency.

The *Pinus* genus, comprising around 250 species, stands as the most extensive genus within the *Pinaceae* family and is known for its antioxidant, anti-inflammatory, anticancer, antifungal, and antimicrobial activities [21]. It grows naturally in many regions of the northern hemisphere, particularly in the Mediterranean, the Caribbean, Asia, Europe, and North and Central America [22]. In the context of co-product valorization (forest residues) and the development of a circular bioeconomy, pine varieties generate large quantities of unvalorized cones (ex. *P. sylvestris* 1.5 × 10^6^ hec, *P. Pinea* 1.43 × 10^6^ hec in France in 2020—IGN). The economic evaluation of pinecones is important in forestry management and cosmetic industrial interest. According to official production data for 2020 from France Bois Forest (https://franceboisforet.fr/, accessed on 26 June 2024), total annual production in France rose to achieve 10^6^ cones per year in state forests. In the Siliana Tunisia forest region, the average yield of pine cones was reported at 160 kg/ha/year in 2015 [23]. In Tunisia, *P. halepensis* produces approximately 112 cones per tree [23] and *P. pinea* yields between 20 and 700 cones per tree [24]. Based on the official annual report in Tunisia (REF 2015), the market value of pinecones was recorded at USD 36 per metric ton in 2015. These findings allow for the estimation of the economic value of pinecones, approximately USD 7 per hectare of forest at 2016 prices. These empirical data highlight the need to explore pinecones and optimize their production, distribution, and utilization. This underscores economic interests not only for forest custodians but also attracts the attention of local, regional, and global economic stakeholders. The potential applications extend across various industries, including pharmaceuticals, cosmetics, and agri-food, indicating promising economic advancement and innovation opportunities.

In research, pinecone extracts have been extensively studied as a promising source of compounds with potential commercial applications in cosmetics, medicine, therapy, and bioconservation [25]. Several approaches have been used to explore alternative and economical uses for pinecones. Researchers have extensively analyzed the chemical composition and isolated extracts from cones of coniferous trees, comparing their findings with those from various types of cones and existing studies to elucidate chemical variances [26]. These insights are instrumental in advancing the utilization of coniferous cone materials as novel pharmaceutical plant products. Conifer cones share a chemical composition similar to coniferous woods, consisting primarily of cellulose, lignin, and hemicellulose. They are rich in phenolic compounds with great biological activity [25]. These cones also contain terpenoids, such as oleoresins, which are the major defense mechanism of conifers. Terpenoids find applications in cosmetics for fragrances, food, and beverages as flavorings, and in traditional medicine for therapeutic purposes like treating diarrhea, coughs, and fever [27]. Furthermore, cones are sources of tannins, including proanthocyanidins, resin acids, and phenolic compounds, as well as stilbenoids [28]. Proanthocyanidins exhibit antioxidant and antimicrobial properties against fungi, bacteria, and viruses in pinecone extracts [25]. Resin acids and tannins extracted from pinecones have potential uses as preservatives and antifungal agents [29]. Stilbenes, such as pinosylvin found in pinecones, possess notable antifungal properties and are utilized in herbal medicines as patented antimicrobial agents [28]. Moreover, essential oils from pinecones containing α-pinene, β-pinene, camphene, and limonene offer therapeutic benefits such as expectorant, antibacterial, antifungal, and antiviral effects. Pine oil is commonly included in preparations for respiratory disease prevention and treatment [30]. The ethyl acetate fraction of pinecones (PEF) demonstrated no toxicity to B16F10 cells at concentrations below 100 μg/mL. PEF inhibited the microphthalmia-associated transcription factor (MITF), tyrosinase, and tyrosinase-related factors in B16F10 cells treated with 3-Isobutyl-1-methylxanthine (IBMX). These findings indicate that pinecones can have the potential as natural agents for inhibiting melanogenesis effectively [31]. In the cosmetic industry, the physical and sensory properties of formulations containing carbonized pine cone powder (CPC) were evaluated, indicating the hydrogel-based ultra-moisturizing cream (HUMC2) was preferred over the lauric acid/Vaseline-based gel (LV2) for spreadability, moistness, and removal capacity [32]. The SFE pinecone extract, investigated by Kim et al. [33], demonstrated high antioxidant and antimicrobial activities, with significant inhibition zones for Staphylococcus aureus and Staphylococcus epidermidis. Additionally, this extract showed over 80% cell viability in cytotoxicity tests, effective anti-inflammatory properties, and high stability in emulsions, demonstrating its potential as a cosmeceutical ingredient for skin regeneration and anti-inflammatory applications.

This study aims to explore the chemical composition and biological activities of *Pinus halepensis*, *Pinus brutia*, and *Pinus pinea* due to their ecological and economic importance in Mediterranean regions. These species are widely distributed and commonly used in traditional medicine and forestry. Previous research has typically examined entire cones. By specifically separating petals and cores from the cones, our goal was to deepen existing knowledge and compare the distinct chemical profiles and biological activities of both plant parts. To our knowledge, the present work is the first to examine the chemical compositions and in vitro antioxidant and anticancer activities of cyclohexane (1SV), ethyl acetate (2SV), and methanol (3SV) extracts from *P. halepensis* (PA), *P. brutia* (PB), and *P. pinea* (PP) cones (both petals (P) and cores (C) fractions) using maceration. The chemical compositions of the eighteen extracts were determined using gas chromatography with mass spectrometry (GC-MS) and high-performance liquid chromatography (HPLC). In vitro antioxidant and anticancer activities were assessed using 1,1-diphenyl-2-picrylhydrazyl (DPPH) and 3-(4,5-dimethylthiazol-2-yl)-2,5-diphenyltetrazolium bromide (MTT) tests.

## 2. Results and Discussion

Extracts derived from various parts of plants play a crucial role in both traditional and modern medicine [34]. It is essential to study forestry residues in a more intensified way to promote the use of residual extracts for determining their potential in agrifood and medicinal applications. Pinecones, including their petal and core fractions, are a type of forestry residue and are known for their medicinal properties.

### 2.1. Extraction Yields

The resulting extraction yields for the eighteen extracts for both fractions P and C from PA, PB, and PP were quantified and reported in Table 1. The highest extraction yield was obtained with PA, followed by PB and finally PP for all extracts (*p* ≤ 0.05). These differences in yield between the different pine species and extraction solvents underline the importance of species selection and solvent choice for the optimization of extraction processes. The fraction P demonstrated the highest yield compared to the fraction C for all samples (*p* ≤ 0.05). The highest yields were reported for 3SV, PA-P-3SV (12.52%), followed by PB-P (8.47%) and PP-P (3.08%), while 1SV showed the lowest yield with PB-C-1SV (0.50%). The yield of the polar fractions (3SV) is higher than that of the apolar fractions (1SV and 2SV) (*p* ≤ 0.05), indicating that the pinecones studied contain more polar compounds than apolar compounds.

Compared to the literature, our results demonstrated a higher extraction yield for P (12.52%) compared to the total cone (10.60% with 3SV) studied by Salim et al. [35], while the yield of C was lower, highlighting the potential benefits of focusing on individual fractions to optimize extraction protocols and increase overall yield. The same study exhibited a significantly higher yield with methanol (10.60%) than hexane (3.80%), indicating the significant effect of polarity. Dhibi et al. [36] used green cones from *P. halepensis* with methanol as solvent and showed a lower extraction yield (2%) than that obtained in this study, illustrating the influence of maturity degree on the extraction process.

### 2.2. Reducing Sugars Content

No prior investigations had been conducted regarding RSC for both P and C from *Pinus*. The RSC values acquired for the eighteen extracts of both P and C from PA, PB, and PP are reported in Table 2. Statistical analysis reveals significant variations in the RSC values among the extracts based on the solvent and species used (*p* ≤ 0.05). PA showed the highest richness in RSC, followed by PB and PP with 3SV (*p* ≤ 0.05). RSC in fraction C was higher than in fraction P for all extracts except PB (*p* ≤ 0.05). 3SV extracts proved the higher RSC than 1SV and 2SV extracts (*p* ≤ 0.05). Among 3SV extract, the highest RSC was observed with PA-C extract showing the highest concentration (594.17 mg/g DW), followed by the PB-P (282.55 mg/g DW), and PP-C (231.37 mg/g DW) (*p* ≤ 0.05). The second highest RSC values were recorded with the 2SV solvent, amounting to 90.69 mg/g DW for PB-P, followed by the 1SV (25.34 mg/g DW) for PB-C. There was a noticeable increase in the concentration of extractable reducing sugars as solvent polarity increased. These results illustrate the richness of pinecones in reducing sugars and the influence of the solvent polarity used in their extraction.

Compared to the literature, Gamli [37] demonstrated that the total cone from *P. brutia* contains between 224.1 and 229.7 mg GE/g DW of the total sugars in the aqueous extract. These results were in the range of RSC obtained in both fractions P and C from PB obtained in our study for polar extract, 3SV.

### 2.3. Total Polyphenol Content

**Figure 1:**
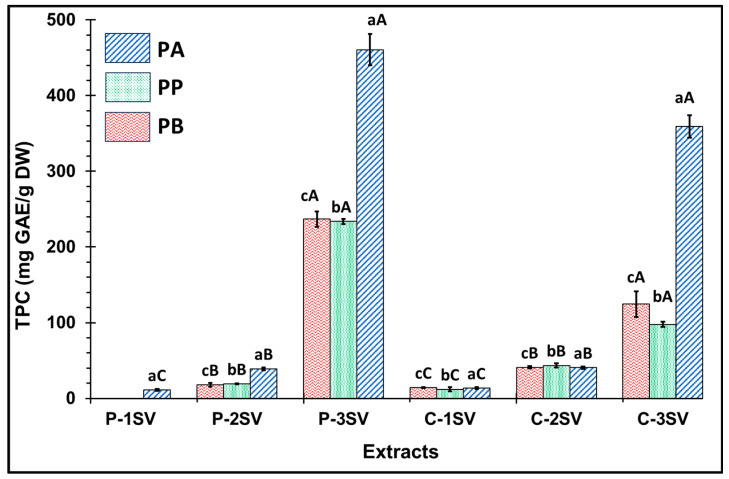
Total phenolic content (TPC) of *P. halepensis*, *P. brutia*, and *P. pinea* (P: petals; C: cores) extracts 1SV: Cyclohexane, 2SV: Ethyl acetate, 3SV: Methanol; GAE: Gallic acid; DW: dry weight. A different letter on the table means a significant difference (*p* ≤ 0.05). Uppercase and lowercase letters refer to solvent and species, respectively. Results are mean ± SD (*n* = 3).

The TPC values were determined for eighteen extracts of PA, PB, and PP, and were illustrated in Figure 1. Statistically, the PA extracts showed higher TPC values than the PB and PP extracts (*p* ≤ 0.05). P fraction demonstrated a higher richness in TPC compared to C fraction (*p* ≤ 0.05). The data analysis showed that polar fractions have a significantly higher TPC concentration than apolar fractions (*p* ≤ 0.05). The 3SV extracts demonstrated the highest TPC values compared to those of the 1SV and 2SV extracts (*p* ≤ 0.05). Methanol was found to be the most effective solvent system for extracting phenolic compounds from different plant parts due to its capability to inhibit polyphenol oxidase activity, thus preventing phenolic compound oxidation [38]. The 1SV extracts showed the lowest TPC values (TPC < 10 mg GAE/g DW). The 2SV extracts showed moderate TPC values ranging from 17.95 to 43.53 mg GAE/g DW. PA-P-3SV and PA-C-3SV were observed to be 460.66 and 359.24 mg GAE/g DW respectively, presenting the highest extracts richness in TPC.

Compared to the literature, the TPC in the total cone from PB-3SV reported by Semerci et al. [39] is the lowest (91 mg GAE/g DW). This discrepancy could be attributed to the influence of the solid–liquid ratio used. Meziti et al. [40] used MeOH/H_2_O (80/20) as a solvent for the extraction of phenolic compounds from the total cone of PA, which yielded the lowest concentration of 251.40 mg GAE/g DW. Costa et al. [41] used ultrasound to extract phenolic compounds from PP-P and PP-C. The results confirm that fraction P has a higher phenolic compound than fraction C (601.8 and 360.6 mg GAE/g DW, respectively) in the ethanolic extract. These values are higher than our results, which may be due to the potential of ultrasound in extracting phenolic compounds.

### 2.4. Antioxidant Activity

Plants serve as a promising natural source of antioxidants, synthesizing antioxidative compounds as a survival mechanism to counteract reactive oxygen species (ROS) [42]. The antioxidant potential of eighteen extracts from PA, PB, and PP was determined using the DPPH assay. Ascorbic acid at 4 µg/mL was used as a reference and demonstrated an inhibition of 72.96% against DPPH. Statistically, the PP showed higher antioxidant potential than PA and PB (*p* ≤ 0.05). The P fraction demonstrated a higher antioxidant activity compared to the C fraction (*p* ≤ 0.05). At 50 µg/mL, PP-P showed a higher percent scavenging activity (94.75%) compared to PA-P (71.17%) and PB-P (63.69%) with 3SV (*p* ≤ 0.05). 3SV extracts showed a significant difference in antioxidant activity (*p* ≤ 0.05) to that of 1SV and 2SV systems. Methanol proved to be an effective solvent system for extracting total phenolic compounds, underscoring the significance of phenolic and polyphenolic compounds as natural antioxidants that enhance free radical scavenging activity [43]. To evaluate the efficacy of 3SV extracts in their antioxidant activity, the Half-maximal inhibitory concentration (IC_50_) was determined from the curve illustrating the relationship between inhibitory activity and concentrations. The lowest IC_50_ value indicates the highest antioxidant potential. As reported in Table 3, PP-P has the best IC_50_ (10.54 µg/mL), followed by PA-P (14.16 µg/mL) and PB-P (26.57 µg/mL) (*p* ≤ 0.05).

These findings are in agreement with previous studies. Meziti et al. [40] found the total cone of PA with MeOH/H_2_O (80/20) extract to have a weaker DPPH inhibitory potential, with an IC_50_ of 18.87 µg/mL. Dhibi et al. [36] showed significantly lower antioxidant activity, with an IC_50_ value of 474 µg/mL for methanolic extracts of the green cone of PA. This discrepancy could be due to the maturation stages of the pinecone. It is well known that the antioxidant properties of plant material can be influenced by factors such as growth stage and environmental conditions. Additionally, Costa et al. [41] confirmed that P has a higher antioxidant potential (IC_50_ = 46.8 µg/mL) than C (IC_50_ = 103.8 µg/mL) when a polar solvent is used.

### 2.5. Anticancer Activity

No prior investigations had been conducted regarding the anticancer activity of PA, PB, and PP extracts. This study marked the first exploration into this aspect. As reported in Table 4, the anticancer activity and toxicity of eighteen extracts, prepared at concentrations of 50 µg/mL, were assessed using three cell lines: two cancer cell lines, a hepatic cancer cell line (HepG2) and human epithelial cervix carcinoma (HeLa), as well as a non-cancerous human embryonic kidney cell line (HEK-293). The MTT test was employed to evaluate both the cytotoxicity of extracts against cancer cells and the toxic effects of extracts on normal cells, ensuring their suitability for potential use in health applications. Tamoxifen, a known cytotoxic agent, was used as a positive control. The results demonstrated a significant decrease in the viability of HepG2, HeLa, and HEK-293 cells when treated with Tamoxifen, thereby validating the accuracy of the experimental method employed (*p* ≤ 0.05).

The most pronounced inhibition of cell growth was achieved by PA, followed by PP and PB with 1SV and 2SV extracts for Hela and HepG2 (*p* ≤ 0.05). However, a moderate to low inhibition potential was detected in 3SV extracts for all species. There are no significant differences in inhibitory effect between P and C. The 1SV exhibited a higher inhibitory effect on Hela cell lines up to 75% for PA and PP, followed by PB with 67.64%, compared to HepG2 cell lines. The same trend was observed with 2SV extracts where the inhibitory potential on Hela was higher than obtained on HepG2. Based on these findings, it appears that HeLa cells exhibit greater sensitivity compared to the HepG2 cell lines. This variation in sensitivity among different cell types has been observed in prior studies [44,45,46].

Compared to the literature, a study conducted by Li et al. [47] focused on the cones of *Pinus yunnanensis*, revealing a notable inhibition rate of 73% against the HepG2 cell line. Moreover, Li et al. [48] evaluated the anticancer activity of *Pinus koraiensis* bark procyanidins extracts against Hela cell lines and obtained an IC_50_ = 196.38 µg/mL.

HEK-293 cells, a non-cancerous cell line, are a transformed cell line originally derived from human embryonic kidney cells through the introduction of sheared adenovirus type 5 DNA fragments. They are classified as immortalized, meaning they have the capability for indefinite cell division [49]. This attribute renders them exceptionally valuable and extensively employed in biomedical research [50]. The evaluation assessed cell viability, proliferation, and any adverse effects induced by the compounds. This analysis provides crucial insights into the safety profile of the compounds (extracts), particularly their impact on non-cancerous cell lines, aiding in assessing their potential therapeutic applications.

The eighteen extracts of PA, PB, and PP revealed a low inhibition value against HEK-293 cell viability at 50 μg/mL, ranging from 0.72 to 11.05%. These values were significantly lower (at least 1/3) than those obtained against both cancer cell lines used (Hela and HepG2).

This observation estimates the nonsignificant toxic effect of our extracts on the viability of healthy normal cells, highlighting the safety and non-toxic characteristics of pinecone extracts from PA, PB, and PP, holding promise for further investigation and potential applications in pharmaceuticals, nutraceuticals, and functional foods.

This aligns with the results of previous studies, which confirmed that pinecones are considered nontoxic and have been used in medicine to moisten lungs, relieve coughing, and reduce fever [51,52]. Pinecones were also a popular folk medicine in Japan, especially for the treatment of gastric cancer [26,53].

### 2.6. Chromatographic Analysis

#### 2.6.1. Identification of Compounds Using HPLC-DAD

The compounds found in both fractions P and C of the extracts of PA, PB, and PP were identified using the HPLC-DAD technique. To determine the composition of the extracts, the retention time (RT) and maximum wavelength (λmax) of each peak were compared with those of reference compounds injected under the same conditions (Table 5). These reference standards were introduced into the system under the same experimental conditions as the *Pinus* extracts.

Phytochemical analysis of extracts resulted in the identification of 38 compounds. These compounds included phenolic compounds, methoxyphenols, and derivatives of p-hydroxybenzoic acid. Twenty-seven compounds have not been previously found in these species, including Gallocyanin, Methyl 3,5-dihydroxybenzoate, Trans-3-hydroxy-cinnamic acid, 5-Hydroxy-4′-methoxylflavone, 4-Hydroxy-3-(3-oxo-1-phenylbu-tyl)coumarine, 3′-Hydroxy-a-naphthoflavone, 7-Hydroxyflavone, Beta carotene, Lutein, 4-Hydroxytamoxifen, 5,7-Dihydroxy-4-propylcoumarine, 3′-hydroxy-6-methylflavone, 5-hydroxyflavone, 3,3′,4′-trimethoxyflavone, Butyl 4-hydroxybenzoate, Cardamonin, Benzyl 4-hydroxybenzoate, 7-Hydroxy-3′,4′,5′-trimethoxy-alpha-naphthoflavone, 3,3′-Dimethoxyflavone, 3,6,3′-Trimethoxyflavone, 3,7-Dimethoxyflavone, 5-Hydroxy-3′-methoxyflavone, Xanthurenic acid, 4′,5′-Dimethoxy-2′-hydroxy-4-methyl-chalcone, (z)-3-(3-Ethoxy-4-hydroxy-phenyl)-2-phenylacrylic acid, Hamamelitannin, and 3,4-Dihydroxy-5-methoxycinnamic acid. The PA and PB extracts revealed the presence of 28 compounds, whereas PP extracts contained 26 molecules. As reported in Table 4, the dominance of these compounds was detected in the P fractions compared to the C fractions of all species. Catechin and chlorogenic acid were exclusively detected in the PA-P-3SV, measuring 59.87 and 56.70, respectively, known for their antioxidant and anticancer properties [54,55]. Gallic acid, a major antioxidant and anticancer commercial standard [54], was identified only in PA-C-3SV and was 131.61. Three molecules found exclusively in PP-P-3SV, Trans-cinnamic acid (1.31), 5-hydroxy-4′-methoxyflavone (0.31), and 4-hydroxy-3-(3-oxo-1-phenylbutyl)coumarin (15.48), are known for their strong antioxidant properties [56]. These molecules are estimated to be responsible for the observed antioxidant activity (10.54 µg/mL) in this extract. The 3-amino-4-hydroxybenzoic acid (ranging from 6.84 to 69.26) and caffeic acid (ranging from 29.93 to 71.50) were detected in 1SV and 2SV of all species studied. This indicates that these compounds play an important role in the chemical properties of these species. In addition, beta-carotene, known for its numerous benefits such as antioxidant and anticancer activities, was found specifically in PB (113.59) and PP (66.63) extracts [57]. Various compounds, including 3′-hydroxy-6-methylflavone, 5-hydroxyflavone, 3,3′,4′-trimethoxyflavone, 3,3′-dimethoxyflavone, 3,7-dimethoxyflavone, 5-hydroxy-3′-methoxyflavone, xanthurenic acid, 4′,5′-dimethoxy-2′-hydroxy-4-methylchalcone, and (z)-3-(3-ethoxy-4-hydroxy-phenyl)-2-phenyl-acrylic acid, were detected in all species studied, suggesting common characteristics between these species.

**Table 5:** Identification of compounds by HPLC-DAD of petals and core extracts from *P. halepensis*, *P. brutia*, and *P. pinea* with different solvents.

Compound	RT min	Structure	Aera mAU·min																		Ref
			PA-C			PA-P			PB-C			PB-P			PP-C			PP-P			
			1SV	2SV	3SV	1SV	2SV	3SV	1SV	2SV	3SV	1SV	2SV	3SV	1SV	2SV	3SV	1SV	2SV	3SV	
Catechin	0.90	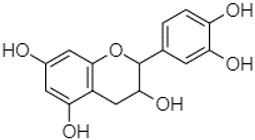	-	-	-	-	-	59.88	-	-	-	-	-	-	-	-	-	-	-	-	[58]
Chlorogenic acid	1.19	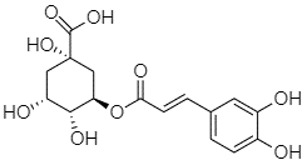	-	-	-	-	-	56.70	-	-	-	-	-	-	-	-	-	-	-	-	[59]
Gallic acid	2.05	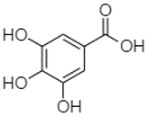	-	-	131.61	-	-	-	-	-	-	-	-	-	-	-	-	-	-	-	[60]
Gallocyanin	2.34	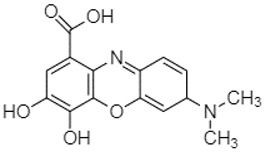	-	-	-	1.11	-	216.16	-	-	-	-	-	73.57	-	-	-	-	-	-	
Methyl 3,5-dihydroxybenzoate	2.52	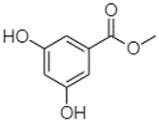	-	2.65	184.98	11.97	34.88	-	-	-	-	-	-	-	-	-	-	-	27.63	-	
3-Amino-4-hydroxybenzoic acid	2.97	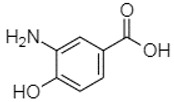	18.89	-	-	-	69.26	-	-	38.31	-	27.80	-	-	6.84	29.93	-	9.54	-	-	[61]
Caffeic acid	3.15	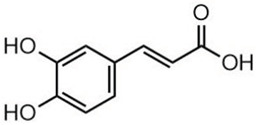	-	-	-	34.76	71.50	-	-	33.54	-	-	29.94	-	-	29.93	40.32	-	-	-	[62]
Sinapic acid	3.33	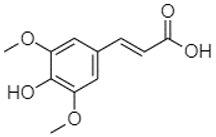	-	-	-	-	79.53	-	-	38.61	42.02	-	24.34	-	-	-	-	-	-	-	[63]
Trans-3-hydroxycinnamic acid	3.65	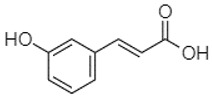	-	-	99.40	-	-	-	-	-	46.89	-	-	-	-	-	-	-	-	60.22	
p-coumaric acid	3.85	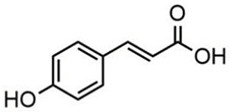	-	-	-	-	-	-	-	-	46.70	-	-	-	-	-	80.78	-	-	-	[63]
Trans-ferulic acid	4.29	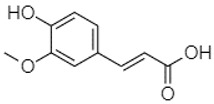	-	-	-	-	-	-	-	-	76.24	-	-	-	-	-	-	-	-	105.09	[62]
Trans-cinnamic acid	10.94	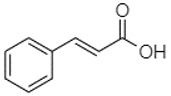	-	-	-	-	-	-	-	-	-	-	-	-	-	-	-	-	-	1.31	[63]
5-Hydroxy-4′-methoxylflavone	18.70	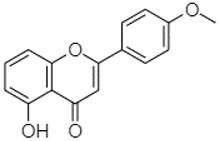	-	-	-	-	-	-	-	-	-	-	-	-	-	-	-	-	-	0.31	
Chrysin	18.92	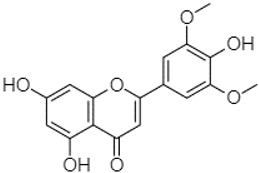	-	-	-	-	-	-	-	-	-	1.58	-	-	-	-	-	-	-	-	[64]
4-Hydroxy-3-(3-oxo-1-phenylbutyl)coumarine	19.10	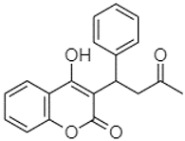	-	-	-	-	-	-	-	-	-	-	-	-	-	-	-	-	-	15.48	
3′-Hydroxy-a-naphthoflavone	19.37	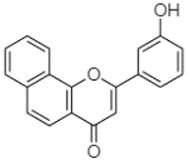	-	21.98	-	-	-	-	-	-	-	90.72	-	-	-	-	-	-	-	-	
7-Hydroxyflavone	19.70	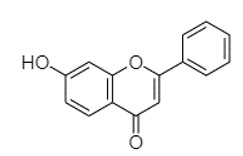	-	-	-	-	17.59	-	-	-	-	-	-	-	-	210.28	-	0.55	24.02	-	
Beta carotene	19.79	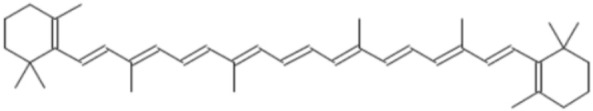	-	-	-	-	-	-	33.59	-	-	5.13	-	113.49	-	-	2.20	66.63	4.75	-	
Lutein	19.81	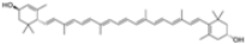	-	-	-	-	3.87	-	-	218.07	-	-	-	-	-	-	26.29	-	-	-	
4-Hydroxytamoxifen	20.28	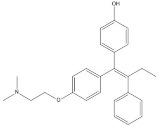	-	-	2.20	11.27	21.10	-	-	355.27	1.11	-	7.74	-	-	84.49	-	2.98	574.05	-	
5,7-Dihydroxy-4-propylcoumarine	20.62	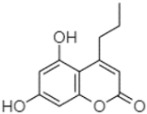	-	-	-	-	-	-	5.98	-	199.33	-	-	-	-	-	-	-	-	-	
3′-hydroxy-6-methylflavone	20.75	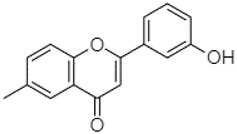	-	17.37	28.50	19.14	57.41	-	-	-	258.76	101.82	8.43	0.79	215.56	-	1.25	52.22	-	250.09	
5-hydroxyflavone	21.00	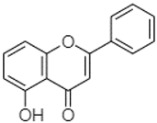	-	-	-	-	-	-	-	-	-	-	-	18.00	-	-	-	126.49	7.21	-	
3,3′,4′_trimethoxyflavone	21.07	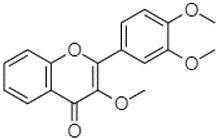	-	-	1.91	118.44	-	-	111.57	-	3.59	105.41	-	-	113.03	208.29	-	72.16	4.99	-	
Butyl 4-hydroxybenzoate	21.12	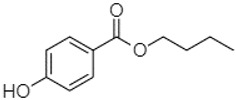	-	179.66	-	-	-	-	-	102.08	-	53.40	491.80	-	-	-	-	-	-	-	
Cardamonin	21.26	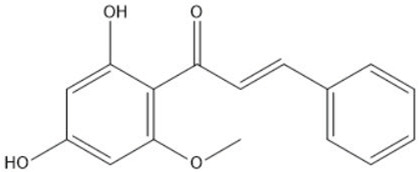	-	-	-	-	-	-	-	-	-	-	-	-	-	-	8.18	-	142.25	-	
Caffeic acid 1,1-dimethylallyl ester	21.34	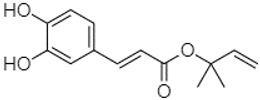	-	220.98	-	-	-	-	-	-	-	2.45	-	-	-	-	2.93	-	-	-	[65]
Benzyl 4-hydroxybenzoate	21.50	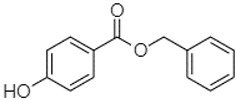	-	-	-	-	20.31	-	73.51	-	-	-	-	-	-	-	-	87.51	-	-	
7-Hydroxy-3′,4′,5′-trimethoxy-alpha-naphthoflavone	21.50	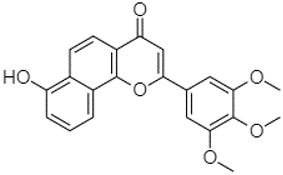	-	8.14	8.67	-	-	-	-	-	82.97	-	-	-	-	-	-	-	-	-	
3,3′-Dimethoxyflavone	21.60	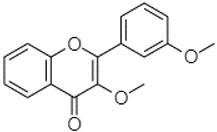	-	-	13.07	90.63	-	-	154.03	-	-	-	-	-	2.53	97.77	-	62.11	-	2.48	
3,6,3′-Trimethoxyflavone	21.79	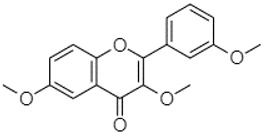	90.08	-	-	-	17.67	3.93	-	-	-	92.96	-	-	-	-	-	-	-	-	
3,7-Dimethoxyflavone	21.83	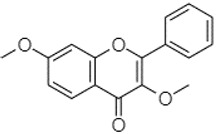	-	-	-	-	212.04	-	-	-	-	-	12.71	-	-	-	-	-	-	-	
5-Hydroxy-3′-methoxyflavone	22.01	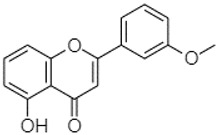	-	-	-	86.86	256.58	-	125.17	-	1.91	81.65	-	6.15	102.77	-	73.07	69.21	7.39	1.95	
Xanthurenic acid	22.12	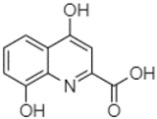	-	17.22	2.35	101.45	-	-	54.68	686.13	-	-	141.72	-	-	32.49	-	-	-	-	
4′,5′-Dimethoxy-2′-hydroxy-4-methylchalcone	22.60	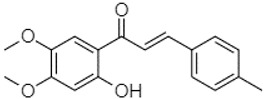	8.46	108.94	13.74	192.08	187.48	-	19.19	-	2.44	243.18	-	-	199.68	-	-	-	11.14	-	
(z)-3-(3-Ethoxy-4-hydroxy-phenyl)-2-phenyl-acrylic acid	23.40	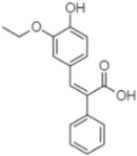	50.13	-	-	10.98	170.16	-	-	246.22	1.61	-	14.01	-	118.02	-	9.06	36.45	-	2.80	
Hamamelitannin	24.06	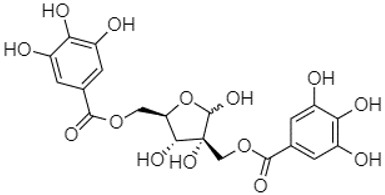	-	-	-	-	-	-	-	-	-	7.95	-	-	14.02	-	8.05	31.36	-		
3,4-Dihydroxy-5-methoxycinnamic acid	25.04	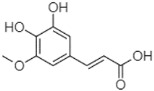	-	-	5.27	64.18	-	-	-	-	-	-	-	-	-	-	-	69.71	5.82		

PA: *Pinus halepensis*; PB: *Pinus brutia*; PP: *Pinus pinea*; P: petal; C: core; 1SV: Cyclohexane; 2SV: Ethyl acetate; 3SV: Methanol; RT: retention time.

#### 2.6.2. Identification of Volatile Compounds by GC-MS

The volatile composition of the different extracts was evaluated by GC-MS analysis.

**Figure 2:**
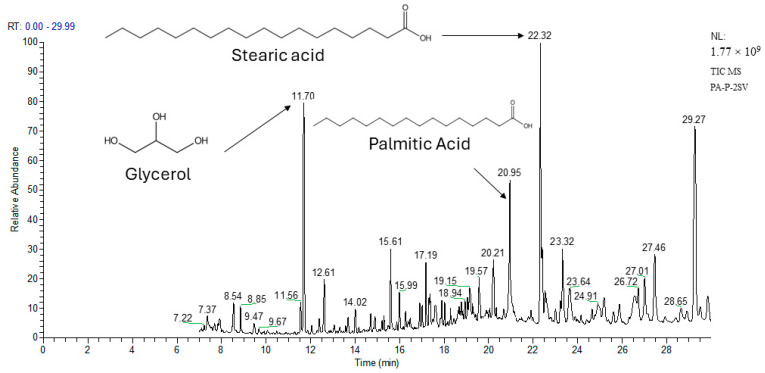
GC–MS chromatogram (Example) of volatile compounds in PA-P-2SV: *P. halepensis* petals for ethyl acetate extract (after derivatization). NL: 1.77 × 10^9^: Normalized Level; TIC: Total ion current; MS: mass spectrometry.

The different structural compounds detected in the extracts before and after derivatization are reported in Table 6 and Table 7, respectively. GC analysis without derivatization was initially performed to identify the volatile compounds present in their natural state. Subsequently, derivatization was used to enhance the detection and identification of additional compounds (for example, polar and dense compounds). Firstly, 22 compounds were initially identified before derivatization. Following derivatization, the number of identified compounds expanded to 45 in the extracts of different species, including polyphenols, organic acids, sugars, ketones, esters, and alcohol. This demonstrates that derivatization significantly improves the analytical method’s sensitivity and accuracy. Thirty-two of the identified volatile compounds were found for the first time in these species, including (+)-Cis-verbenyl acetate, isobornyl formate, p-Cymen-8-ol, carveol, isobornyl acetate, Bicyclohexyl, Tetradecane, Bicyclo [2.2.1]heptane-2,5-diol, 1,7,7-trimethyl-, (2-endo,5-exo)-, Limonene glycol, 2,4-Di-tert-butylphenol, phenol, 2,2′-methylenebis [6-(1,1-dimethylethyl)-4-methyl-, 18-Norabieta-8,11,13-triene, Sclareol, dehydroabietin, 17-Pentatriacontene, 7-Isopropyl-1,4a-dimethyl-1,2,3,4,4a,9,10,10a-octahydro-1-phenanthrenol (isomer 1), Dehydroabietal, oleamide, glycol, propyl glycol, cyclohexanol, caproic acid, 3-Hydroxybutyric acid, (+)-Cis-verbenol, glycerol, caprylic acid, (-)-Myrtenol, succinic acid, pelargonic acid, cicrotoic acid, myrtenoic acid, and D-(-)-Ribofuranose. The PA extracts revealed the presence of 42 compounds, whereas the PB and PP extracts contained 30 and 27 molecules, respectively. The dominance of these compounds was detected in the P fractions compared to the C fractions of all species. Figure 2 illustrates an example of the GC-MS machine-generated graph depicting the retention time of the richest extract, PA-P-2SV by volatile compounds. Intriguingly, nine compounds frequently found in all extracts were shared between the extracts of studied species, such as verbenone, carveol, 2,4-di-tert-butylphenol, oleamide, propyl glycol, cyclohexanol, caproic acid, myrtenic acid, and palmitic acid. Their presence in these extracts could provide valuable insights into the chemistry of these plant species and their potential applications in various fields. The minority identification of compounds in the methanolic extracts by GC-MS analysis raises important questions about the chemical nature of the molecules present. GC-MS is known for its ability to analyze volatile or semi-volatile compounds, but it can pose limitations when dealing with larger or highly polar molecules. PA, PB, and PP demonstrated their richness in organic acids as indicated by their substantial surface area, with a predominance of both protocatechuic acid and palmitic acid ranging from 106 to 681 x10^7^. Previous studies have identified these two compounds as being responsible for antimicrobial, antioxidant, and anti-inflammatory activities [66]. Nine compounds were detected specifically in PA which present a high surface area such as isobornyl formate (2.58 × 10^7^), 17-pentatriacontene (23.23 × 10^7^), dehydroabietin (22.99 × 10^7^), 3-hydroxybutyric acid (10.06 × 10^7^), vanillin (33.1 × 10^7^), D-(-)-ribofuranose (69.8 × 10^7^), vanillic acid (29.1 × 10^7^), p-coumaric acid (20.48 × 10^7^), and caffeic acid (784 × 10^7^), providing the specificity of PA into the bioactivities.

These molecules are known for their antioxidant, anti-inflammatory, anticancer, and antimicrobial properties [66]. Previous studies have extensively studied p-coumaric acid isolated from plants for their bioactivities in the pharmaceutic industry. It has been demonstrated that it is a phenolic acid with low toxicity in mice (LD_50_ = 2850 mg/kg body weight) and acts as a precursor to other phenolic compounds [67]. These studies have highlighted the significant biological activities of p-coumaric acid and its conjugates, including antioxidant, anticancer, antimicrobial, antiviral, and anti-inflammatory effects. Furthermore, they have demonstrated its potential in mitigating diabetes, obesity, hyperlipidemia, and gout [67]. Moreover, Ghareib et al. [68] have demonstrated that vanillic acid, identified and isolated from the active acetone fraction of *Chenopodium murale*, has significant antioxidant properties and reduces oxidative stress at low concentrations. In addition, the study of Jaskiran Kaur [69] confirmed that vanillic acid isolated from plants exerts diverse bioactivities against cancer, diabetes, obesity, and hepatic diseases by inhibiting associated molecular pathways. Due to these benefits, vanillic acid has great potential to be used as a nutraceutical and offers scope for therapeutic applications beyond its traditional use as a flavoring agent [69]. Therefore, observed antioxidant and anticancer activities in the PA extract could contribute to the presence of vanillic acid.

Compared to the literature, Fekih et al. [70] revealed the identification of 49 volatile compounds through GC-MS analysis in various parts of *P. halepensis*, including needles, twigs, and buds, revealing the presence of caryophyllene oxide and bornyl acetate. Furthermore, Dhibi et al. [36] identified palmitic acid and stearic acid in *Pinus halepensis* seeds and cones. Pasqualini et al. [71] revealed the identification of various compounds, including protocatechuic acid, vanillic acid, p-coumaric acid, 4-hydroxybenzoic acid, vanillin, syringic acid, and gallic acid.

**Table 6:** Identification of volatile compounds before derivatization by GC-MS of *P. halepensis*, *P. brutia*, and *P. pinea* petals and cores extracts.

Compound	RT min	Structure	Area (×10^7^)																		Ref
			PA-C			PA-P			PB-C			PB-P			PP-C			PP-P			
			1SV	2SV	3SV	1SV	2SV	3SV	1SV	2SV	3SV	1SV	2SV	3SV	1SV	2SV	3SV	1SV	2SV	3SV	
Isopinocarveol	9.04	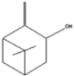	-	-	-	13	-	-	8.49	0.2	-	-	0.11	-	-	-	-	-	-	-	[72]
(+)-Cis-verbenyl acetate	10.84	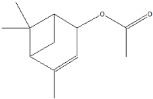	-	-	-	5.3	-	-	-	-	-	-	-	4.61	-	-	-	-	-	-	
Isobornyl formate	11.39	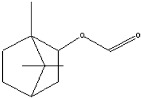	2.58	-	-	-	1.28	-	-	-	-	-	-	-	-	-	-	-	-	-	
p-Cymen-8-ol	11.82	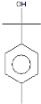	-	3.97	-	-	-	-	-	-	-	-	3.6	-	-	-	-	-	-	-	
Carveol	12.27	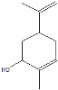	6.87	4.44	-	-	1.83	-	-	-	-	-	-	4.42	19.6	13.6	-	7.3	8.39	-	
Verbenone	12.58	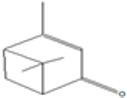	75.7	36.1	0.98	24	15.7	1.59	40.4	27	-	61.8	33.3	6.82	24	-	-	35.8	16.1		[73]
Isobornyl acetate	13.27	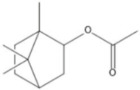	17	8.45	-	8.42	-	-	-	-	-	-	-	-	-	-	-	7.21	-	-	
Bicyclohexyl	13.43	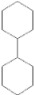	15.8	-	-	21	-	-	20.8	-	-	7.45	-	-	19.4	-	-	10.4	-	-	
Tetradecane	13.87	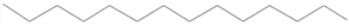	-	-	-	-	-	-	-	-	-	1.64	-	-	-	-	-	-	-	-	
Bicyclo [2.2.1]heptane-2,5-diol, 1,7,7-trimethyl-, (2-endo,5-exo)-	14.19	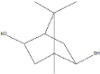	-	-	-	-	-	-	-	0.5	-	-	-	-	-	-	-	-	0.9	-	
Limonene glycol	14.49	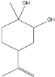	-	-	-	-	-	0.3	-	-	-	-	-	25.4	45	61.4	-	12.6	13.7	-	
2,4-Di-tert-butylphenol	16.33	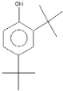	-	5.42	12.6	-	10.8	18.7	-	4.67	-	-	3.22	19.4	-	5.73	15	-	3.58	-	
Caryophyllene oxide	17.62	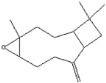	18.9	-	-	-	-	-	-	-	-	-	-	-	-	-	-	51.8	-	-	[74]
Phenol, 2,2′-methylenebis [6-(1,1-dimethylethyl)-4-methyl-	17.92	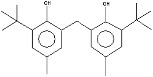	-	-	-	-	74.7	-	-	-	-	-	-	-	-	100	-	-	-	-	
18-Norabieta-8,11,13-triene	21.86	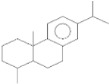	8.61	9.63	-	7.58	4.05	-	-	-	-	-	3.67	-	-	-	-	0.1	-	-	
Sclareol	21.97	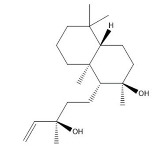	-	-	-	6.13	-	-	-	-	-	52.5	10.6	-	6.54	-	-	23.1	-	-	
dehydroabietin	22.99	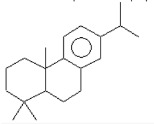	-	12.2	-	11	11.7	-	-	-	-	-	8.54	-	-	-	-	7.25	-	-	
17-Pentatriacontene	23.23	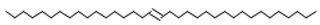	-	-	-	-	19.5	-	-	-	-	-	-	-	-	-	-	-	-	-	
7-Isopropyl-1,4a-dimethyl-1,2,3,4,4a,9,10,10a-octahydro-1-phenanthrenol (isomer 1)	25.11	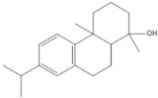	-	8.95	-	10.7	8.77	-	9.25	4.65	-	45.1	6.79	-	-	-	-	-	-	-	
Dehydroabietal	27.98	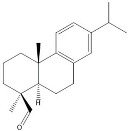	-	-	-	68.5	0.1	-	-	-	-	-	-	-	-	-	-	-	-	-	
Oleamide	28.26	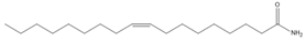	109	-	45.6	-	133	59.4	-	174	-	47.4	-	-	-	-	35.1	-	-	-	
Methyl dehydroabietate	29.23	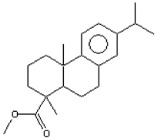	15.2	-	-	204	-	-	-	-	-	-	-	-	-	-	-	-	15.8	-	[75]

PA: *Pinus halepensis*; PB: *Pinus brutia*; PP: *Pinus pinea*; P: petal; C: core; 1SV: Cyclohexane; 2SV: Ethyl acetate; 3SV: Methanol; RT: retention time.

**Table 7:** Identification of volatile compounds after derivatization by GC-MS of *P. halepensis*, *P. brutia*, and *P. pinea* petals and cores extracts.

Compound	RT min	Structure	Area (×10^7^)																		Ref
			PA-C			PA-P			PB-C			PB-P			PP-C			PP-P			
			1SV	2SV	3SV	1SV	2SV	3SV	1SV	2SV	3SV	1SV	2SV	3SV	1SV	2SV	3SV	1SV	2SV	3SV	
Glycol	7.13	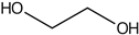	-	7.98	-	-	11.8	-	-	7.39	-	-	6.26	-	-	-	-	-	-	-	
Propyl glycol	7.29	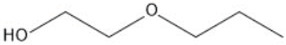	7.56	26.4	-	10	46.7	-	39.1	-	-	53.7	92.3	-	35.3	-	-	0.05	-	-	
Cyclohexanol	7.86	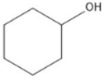	94.1	-	-	202	-	-	244	-	-	223	-	-	249	-	-	93.1	0.07	-	
Lactic acid	8.74	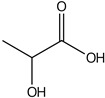	-	0.1	-	-	58	-	-	103	-	-	52.1	-	-	55.8	-	-	-	-	[76]
Caproic acid	8.84	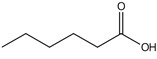	207	43.9	-	58.9	32.3	-	136	37.3	-	-	21.1	-	-	43.3	-	-	3.57	-	
3-Hydroxybutyric acid	10.06	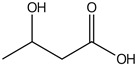	-	-	-	-	3.62	-	-	-	-	-	-	-	-	-	-	-	-	-	
(+)-Cis-verbenol	11.55	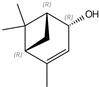	-	7.65	-	-	6.89	-	-	-	-	-	-	-	-	-	-	27.3	-	-	
Glycerol	11.7	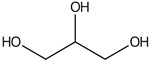	-	35.1	-	-	80.4	-	-	-	-	-	-	-	-	51.2	-	-	-	-	
Caprylic acid	12.05	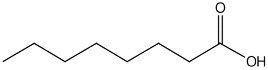	38.3	-	-	-	15.4	-	-	-	-	-	-	-	-	-	-	24.4	-	-	
(-)-Myrtenol	12.84	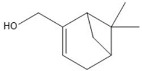	-	-	-	-	-	-	48.1	-	-	50.8	-	-	-	-	-	-	-	-	
Succinic acid	13.06	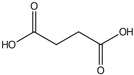	-	-	-	-	11.2	-	-	-	-	-	14.1	-	-	-	-	-	-	-	
Pelargonic acid	13.57	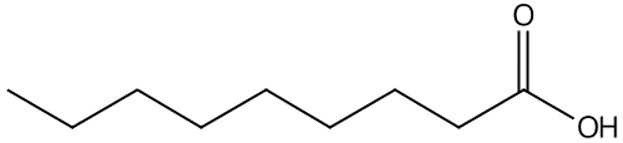	54.9	-	-	5.01	8.37	-	-	-	-	-	-	-	-	-	-	28.5	-	-	
Citric acid	14.01	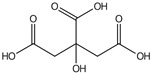	-	-	-	-	-	-	-	84.1	-	-	-	-	-	-	-	-	0.41	-	[76]
Cicrotoic acid	14.17	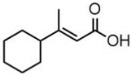	-	-	-	2.14	-	-	3.47	-	-	-	-	-	-	-	-	-	-	-	
Myrtenoic acid	14.89	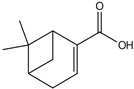	24.5	-	-	6.94	10.1	-	125	14.2	-	215	57.5	-	-	-	-	32.7	-	-	
Vanillin	16.93	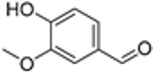	-	33.1	-	-	-	-	-	-	-	-	-	-	-	-	-	-	-	-	[77]
D-(-)-Ribofuranose	17.22	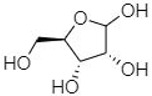	-	11.2	-	-	69.8	-	-	-	-	-	-	-	-	-	-	-	-	-	
Vanillic Acid	18.82	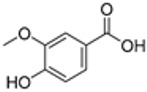	-	-	-	-	29.1	-	-	-	-	-	-	-	-	-	-	-	-	-	[78]
Protocatechuic acid	19.1	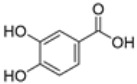	-	106	-	-	181	-	-	187	-	-	-	-	-	-	-	-	200.2	-	[78]
p-Coumaric acid	20.48	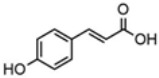	-	9.08	-	-	5.51	-	-	-	-	-	-	-	-	-	-	-	-	-	[78]
Palmitic Acid	20.94	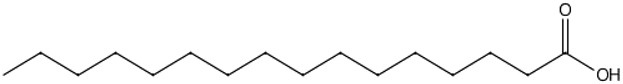	134	321	-	-	307	-	495	681	-	185	200.1	-	335	528	-	270.7	205	-	[79]
Caffeic acid	21.96	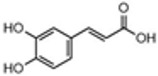	7.54	-	-	-	784	-	-	-	-	-	-	-	-	-	-	-	-	-	[78]
Stearic acid	23.35	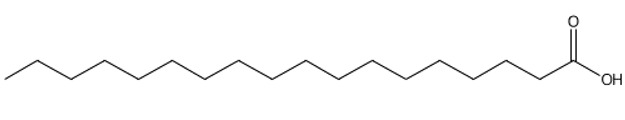	-	42.3	-	-	-	-	39.7	-	-	-	184	-	-	-	-	-	17.9	-	[79]

PA: *Pinus halepensis*; PB: *Pinus brutia*; PP: *Pinus pinea*; P: petal; C: core; 1SV: Cyclohexane; 2SV: Ethyl acetate; 3SV: Methanol; RT: retention time.

### 2.7. Principal Component Analysis (PCA)

**Figure 3:**
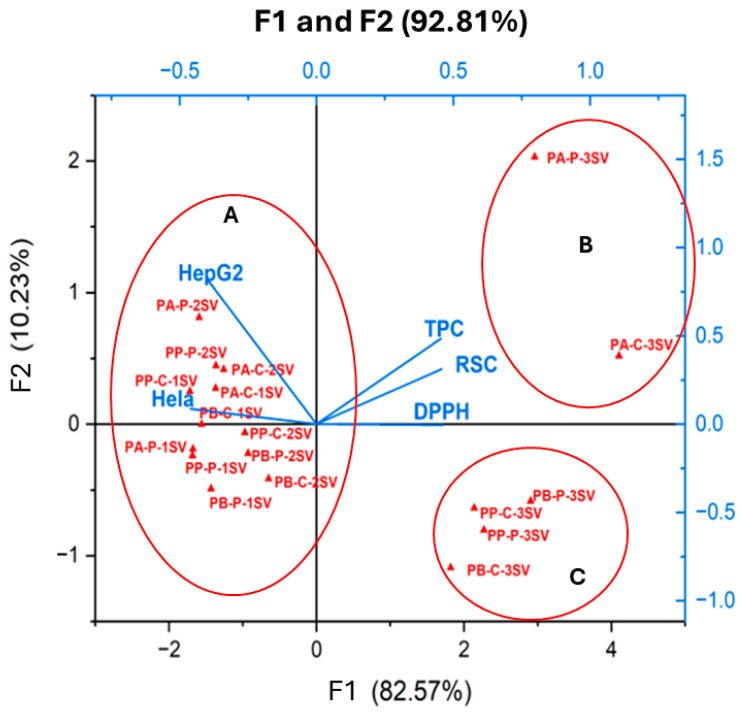
Principal component analysis of TPC: total phenolic content, RSC: Reducing sugar content, DPPH: Antioxidant activity and HepG2 and Hela: anticancer activity from *P. halepensis* (PA), *P. brutia* (PB) and *P. pinea* (PP) of both petals (P) and cores (C) extracts. 1SV: Cyclohexane; 2SV: ethyl acetate; and 3SV: Methanol.

To gain a deeper insight into the relationship between TPC, RSC, and the bioactivities assessed for the PA, PB, and PP extracts, a principal component analysis (PCA) was employed. As reported in Table 8 and Figure 3, this analysis sought to elucidate the connections among five key components, namely TPC (total polyphenolic compounds), RSC (reducing sugars content), % inhibition DPPH (antioxidant activity against DPPH), % inhibition Hela (anticancer activity against Hela cell line), and % inhibition HepG2 (anticancer activity against HepG2 cell line) for the three plant materials. As illustrated in Figure 3, the first two principal components (F1 and F2) encompassed a substantial 92.81% of the data variability for PA, PB, and PP. The primary axis (F1) was strongly positively correlated with TPC, antioxidant activity, and RSC with correlation coefficients R^2^ of 0.86, 0.83, and 0.92, respectively. F2 was only correlated with the anticancer activity. As shown in Figure 3, eighteen extracts exhibit clear segregation into three principal groups (A, B, and C). Group A comprises two extracts (PA-P-3SV and PA-C-3SV), group B includes four extracts (PP-P-3SV, PP-C-3SV, PB-P-3SV, and PB-C-3SV), and group C encompasses the remaining extracts. Notably, extracts in group A were characterized by the highest TPC, RSC, and antioxidant activity. Group B represents the remaining methanolic extracts of PB and PP, characterized by high TPC, RSC, and antioxidant activity but with less significance compared to group A. Group C encompasses extracts in cyclohexane and ethyl acetate from all species, demonstrating interesting activity against Hela and HepG2 cell lines. This grouping highlights the varying bioactivity profiles of the extracts from different pine species and fractions.

Numerous studies have explored the correlation between polyphenol content and antioxidant activity in plant extracts. In this regard, the study of Ait Atmane et al. [80] found a substantial correlation (R^2^ = 0.95) between TPC and DPPH in *P. halepensis* seeds.

## 3. Materials and Methods

### 3.1. Chemicals

All chemicals used were of analytical reagent grade. All reagents were obtained from Sigma Aldrich (Saint-Quentin, France): Cyclohexane, ethyl acetate, methanol, DMSO, DPPH, DNSA, Folin–Ciocalteu reagent (2N), gallic acid, HCl, KH_2_PO_4_, MTT, NaOH, Na_2_HPO_4_, sodium carbonate, tamoxifen.

### 3.2. Collection and Identification of Plant Materials

The mature and naturally dried cones of *P. halepensis* (PA), *P. brutia* (PB) and *P. pinea* (PP) (family *Pinaceae*) were collected in Bizerte (altitude: 37°16′27″, longitude: 9°52′26″, northern Tunisia) in December 2016, and identified by Dr. Hamrouni Lamia and stored at the National Institute of Research on Rural Engineering, Water and Forests (INRGREF) in Ariana, Tunisia.

### 3.3. Sample Preparation

The pinecones were divided into two fraction petals (P) and cores (C), which were separated manually. Grinding was carried out in a standardized two-step process. In the first step, a SACEM hammer mill (model: G8042, power: 5.5 kW, frequency: 50 Hz, speed: 1450 rpm) with a 13 mm sieve was used. The grinding time for cores (C) and petals (P) was approximately 30 min. In the second step, a knife mill (FRITSCH Pulverisette 19) was used with five knives (frequency: 50–60 Hz and 2100 watts). A 1 mm mesh was used and the comminution times were between 24 and 31 min.

### 3.4. Extraction of Extracts

Extracts from PA, PB, and PP, as well as P and C fractions, were extracted through maceration. Five grams of each sample were subjected to continuous extraction for two hours at 20 °C, using moderate stirring and organic solvents with increasing polarity: Cyclohexane (1SV), ethyl acetate (2SV), and methanol (3SV), with a sample-to-solvent ratio of 1:10 (*w*/*v*) under ambient pressure and temperature. The filtered extracts were concentrated by distillation in a rotary evaporator (IKA, RV 10 auto V, Germany) under vacuum at reduced pressure and a temperature of 35 °C.

The extraction yield was determined using the following Equation (1):

(1) $$\%\;yield=\frac{m_{DW}}{m_{dm}}\times 100$$

where $m_{DW}$ represents the weight of dry extract (g) and $m_{dm}$ denotes the weight of dry plant material (g).

### 3.5. Quantification of Reducing Sugar Content

Quantification of reducing sugars (RSC) in extracts 1SV, 2SV, and 3SV of PA, PB, and PP for both fractions P and C was performed according to the 3,5-dinitrosalicylic acid (DNSA) method as described by Ayadi et al. [81], with minor modifications. A total of 150 μL of each extract (350 mg/L) was mixed with 150 μL of DNS solution. After incubation at 100 °C for 5 min with constant stirring, 750 μL of deionized water was added. Then, the absorbance of the mixture was measured at 530 nm. This measurement was performed with a reference blank containing solvent (sodium potassium tartrate in NaOH 2 M) instead of DNSA and a negative control in which the extract was replaced by dimethyl sulfoxide (DMSO). The sugar content was determined in milligrams of glucose equivalent per gram of dry extract (mg GAE/g DW).

### 3.6. Quantification of Total Phenolic Content (TPC)

The total phenolic content (TPC) of different obtained 1SV, 2SV, and 3SV of PA, PB, and PP for both fractions P and C was estimated with a colorimetric assay using the Folin–Ciocalteu method as described by Ayadi et al. [81]. In a basic environment generated by the sodium carbonate (Na_2_CO_3_) and upon oxidation of the sample’s phenols, the Folin–Ciocalteu reagent’s phosphotungstic acid and phosphomolybdic acid were reduced to a blue-colored complex, which was proportional to the number of phenolic compounds present. The blue color intensity was assessed with a microplate reader (Multiskan Go, F1-01620, Thermo Fisher Scientific, Vantaa, Finland) at 765 nm. TPC content was reported as milligrams of gallic acid equivalents per gram of dry weight (mg GAE/g DW) using the regression equation derived from the standard calibration curve of known gallic acid concentrations (0 to 115 mg/L).

### 3.7. Determination of Antioxidant Activity

The free radical scavenging activity of the different extracts 1SV, 2SV, and 3SV of PA, PB, and PP for both fractions P and C was established by Ayadi et al. [81] using the 1,1-diphenyl-2-picrylhydrazyl (DPPH) test. In a 96-well microplate (Micro Well; Thermo Fisher Scientific, Illkirch, France), 20 µL of each diluted extract (0.5 mg/mL) was combined with 180 µL of a methanolic DPPH solution (0.2 N). Afterward, the reaction mixture was incubated for 25 min at 25 °C. A microplate reader (Multiskan Go F1-01620, Thermo Fisher Scientific, Vantaa, Finland) was then used to measure absorbance at 524 nm. In this test, the ascorbic acid was used as a reference at 4 µg/mL.

The percentage inhibition of DPPH was determined by the following Equation (2):

(2) $$\%\;inhibition=100\times (\frac{A_{blank}-A_{sample}}{A_{blank}})$$

The “*A_blank_*” represents the absorbance of the solvent and DPPH radical when no samples are present, and the “*A_sample_*” represents the absorbance of the sample and DPPH radical.

### 3.8. Determination of Anticancer Activity

The anticancer activity of the extracts 1SV, 2SV, and 3SV of PA, PB, and PP for both fractions P and C was estimated on both cancer cell lines: a human liver cancer cell line (HepG2) and human epithelial cervix carcinoma (Hela). Their toxicity effect was also assessed in human embryonic kidney cells (HEK-293). All were purchased from the American Type Culture Collection (ATCC, Manassas, VA, USA). HepG2 and Hela cell lines were cultured in DMEM (Advanced DMEM, Thermo Fisher Scientific) and HEK-293 cell lines were cultured in high-glucose DMEM (Dulbecco’s Modified Eagle’s Medium, France). Each growth medium was supplemented with 10% decomplemented fetal bovine serum, 1% non-essential amino acids, and antibiotics including penicillin, streptomycin, and gentamicin. Cell cultures were maintained in a humidified incubator at 37 °C with 5% carbon dioxide (CO_2_). Upon reaching 70–80% confluence, the cells were harvested and used for conducting cytotoxicity assays. Adherent cells were seeded at a density of 12,000 cells/well in a 96-well microplate for HepG2, Hela, and HEK-293. The microplate was subsequently incubated overnight at 37 °C in a thoroughly humidified atmosphere with 5% CO_2_. Following that, the cells were treated in triplicate with each diluted extract at 50 µg/mL and incubated for 48 h at 37 °C. To evaluate cytotoxicity, we employed the 3-(4,5-dimethyl-thiazol-2-yl)-2,5-diphenyltetrazolium bromide (MTT) test as described by Ayadi et al. [81]. After removing the supernatant, cells received 50 μL of MTT solution and were incubated at 37 °C for 40 min. Following incubation, the MTT solution was removed, and the resulting dark-blue formazan crystals, generated by the reduction in the yellow soluble MTT through mitochondrial dehydrogenase enzymes in viable cells, were dissolved in 80 μL of DMSO. The absorbance at 605 nm was then measured using a microplate reader (Mullikan Go, F1-01620, Thermo Fisher Scientific, Vantaa, Finland). Tamoxifen at 1, 10, and 100 μM was used as a reference in this test.

### 3.9. Identification of Bioactive Compounds

#### 3.9.1. High-Performance Liquid Chromatography (HPLC-DAD)

The analysis of the extracts 1SV, 2SV, and 3SV of PA, PB, and PP for both fractions P and C was performed using an HPLC-DAD system consisting of a Thermo Scientific Accela pump equipped with an Accela PDA detector as described by Ben Khadher et al. [82]. Compound detection was selected at 280 nm. The separation was conducted using an RP-C18 column (Phenomenex; Le Pecq, France) with dimensions of 25 cm × 4.6 mm and a particle size of 5 μm. Elution was carried out at a flow rate of 0.5 mL/min. The mobile phase consisted of acidified water (pH = 2.65) as solvent A and a mixture of acidified water/ACN (20/80 *v*/*v*) as solvent B. A linear gradient elution method was employed: the concentration of solvent B increased from 12 to 30% over 15 min, then further rose from 30 to 50% within 2 min, and finally reached 99.9% in 3 min. Subsequently, it was returned to 12% B in 7 min. The extracts were dissolved in a solution consisting of acidified water/ACN (20/80 *v*/*v*) at 10 mg/mL. Subsequently, they were filtered using a 0.2 μm Sigma Aldrich Millex-HA filter (Saint-Quentin-Fallavier, France). Compound identification was based on the comparison of their retention times and lambda max values with established reference standards.

#### 3.9.2. Gas Chromatography-Mass Spectrometry (GC-MS)

The volatile composition of the extracts 1SV, 2SV, and 3SV of PA, PB, and PP for both fractions P and C was analyzed according to the method described by Ben Khadher et al. [82] with modifications. The extracts obtained were dissolved in their respective extraction solvents at a concentration of 3 mg/mL. The analysis was conducted using a Saturn 2000 gas chromatograph (Les Ulis, France) equipped with a fused silica capillary DB-5MS column (5% phenylmethylpolysiloxane, 30 × 0.25 mm, with a film thickness of 0.25 μm). Hydrogen gas was employed as the carrier gas in this analytical procedure. The column oven temperature program followed this sequence: starting at 60 °C, it was maintained for 1 min, then gradually increased at a rate of 10 °C per min until it reached 150 °C. It was held isothermally at 150 °C for 1 min. Subsequently, another gradient was applied to reach 260 °C at a rate of 12 °C per min and then held at 260 °C for 10 min. For mass spectrometry, each acquisition recorded data in full-scan mode within the range of 70 to 800 AMU. The ion source was maintained at 220 °C, and the transfer line was heated to 240 °C. An injection volume of 5 µL was used for each extract. Compound identification in the extracts was accomplished by comparing their mass spectra with those available in the NIST08 database (National Institute of Standards and Technology, https://www.nist.gov/, MS library version 2.4, build 25 March 2020).

Derivatization method: The derivatization procedure consisted of taking 290 μL of the samples prepared as described above and adding 60 μL of N, O bis(trimethylsilyl)trifluoroacetamide (BSTFA) reagent. This mixture was then incubated at 40 °C for 30 min. Subsequent spectral analysis of each derivative solution followed the procedure described in the previous section. Derivatization is a crucial technique in analytical chemistry used to increase sensitivity and accuracy, especially in GC and GC-MS. It involves modifying analytes or samples with specific chemicals to improve their ability to separate and detect them. This process selectively alters analytes without significantly affecting the sample matrix. For instance, derivatization replaces active hydrogens (typically polar and non-volatile) in functional groups like OH, COOH, SH, NH, and CONH. This modification makes these compounds more volatile, essential for their analysis by GC or GC-MS, where volatility is critical for accurate measurement and identification [83].

### 3.10. Statistical Analysis

The experimental data were expressed as mean values with standard deviations, and each sample was measured in triplicate. The difference between the solvents used and the different pinecone species was evaluated using the Tukey test. In addition, principal component analysis (PCA) was performed using XLSTAT (version 2021.3.1, Addinsoft, Pearson edition, Waltham, MA, USA).

## 4. Conclusions

This research project provided a comprehensive exploration into the chemical compositions and bioactivities of extracts obtained from three different Pinus species—*P. halepensis*, *P. brutia*, and *P. pinea*—collected from Tunisia. The use of HPLC-DAD analysis helped uncover twenty-seven previously unknown compounds in the species studied. These compounds included phenolic compounds, methoxyphenols, and derivatives of p-hydroxybenzoic acid. The use of GC-MS analysis uncovered forty-six volatile compounds, of which thirty-two were detected for the first time in this species. In terms of antioxidant potential, 3SV extracts exhibited significant activity against DPPH. Moreover, the 1SV and 2SV extracts exhibited interesting anticancer activity at a concentration of 50 μg/mL. Notably, all extracts from PA, PB, and PP showed no negative effects on the viability of healthy normal cells (HEK-293 cell line).

Based on these observations, all studied extracts underscore their safety and non-toxic properties, suggesting significant potential for applications in pharmaceuticals, nutraceuticals, and cosmetics. These findings motivate further investigation to isolate the specific molecule responsible for their bioactivities. Furthermore, in vivo and clinical trials will be used to explore their therapeutic potential in pharmaceutical contexts. These steps are crucial for the development of new therapeutic agents with broad-ranging applications in the pharmaceutical and cosmetic industries.

## Figures and Tables

**Table 1:** Extraction yields (%) of bioactive compounds from *P. halepensis*, *P. brutia*, and *P. pinea* petals and cores with different solvent extracts.

Yields (%)			
Fractional Extraction	1SV	2SV	3SV
PA-P	4.30	5.50	12.52
PA-C	2.30	1.90	3.87
PB-P	1.20	4.59	8.47
PB-C	0.50	2.54	2.84
PP-P	1.13	3.00	3.08
PP-C	0.70	1.60	1.18

PA: *Pinus halepensis*; PB: *Pinus brutia*; PP: *Pinus pinea*; P: petal; C: core; 1SV: Cyclohexane; 2SV: Ethyl acetate; 3SV: Methanol.

**Table 2:** Reducing sugar content (RSC) (mg GE/g DW) in *P. halepensis*, *P. brutia*, and *P. pinea* petals and cores with different solvent extracts.

RSC (mg GE/g DW)			
Fractional Extraction	1SV	2SV	3SV
PA-P	10.86 ± 2.09 ^aC^	70.93 ± 2.37 ^aB^	367.24 ± 1.48 ^aA^
PA-C	21.84 ± 1.62 ^aC^	82.59 ± 3.97 ^aB^	594.17 ± 5.08 ^aA^
PB-P	19.09 ± 2.72 ^bC^	90.69 ± 9.70 ^bB^	282.55 ± 9.31 ^bA^
PB-C	25.34 ± 4.79 ^bC^	87.02 ± 3.88 ^bB^	144.18 ± 9.01 ^bA^
PP-P	7.96 ± 1.14 ^cC^	70.72 ± 3.85 ^cB^	201.32 ± 8.93 ^cA^
PP-C	21.77 ± 4.30 ^cC^	80.87 ± 4.08 ^cB^	231.37 ± 7.94 ^cA^

PA: *Pinus halepensis*; PB: *Pinus brutia*; PP: *Pinus pinea*; P: petal; C: core; 1SV: Cyclohexane; 2SV: Ethyl acetate; 3SV: Methanol; GE: glucose equivalent; DW: dry weight. A different letter on the table means a significant difference (*p* ≤ 0.05). Uppercase and lowercase letters refer to solvent and species, respectively. Results are mean ± SD (*n* = 3).

**Table 3:** IC_50_ of Antioxidant activity of petals and cores extracts from *P. halepensis*, *P. brutia*, and *P. pinea* with methanol solvent.

Fractional Extraction							
3SV Extracts							
	PA-P	PA-C	PB-P	PB-C	PP-P	PP-C	Ascorbic Acid
DPPH IC_50_ (µg/mL)	14.16 ± 2.25 ^b^	16.63 ± 0.67 ^b^	26.57 ± 0.95 ^d^	50.01 ± 3.74 ^e^	10.54 ± 0.24 ^a^	22.46 ± 1.82 ^c^	3.56 ± 0.35

PA: *Pinus halepensis*; PB: *Pinus brutia*; PP: *Pinus pinea*; P: petal; C: core; 3SV: Methanol; IC_50_: Half-maximal inhibitory concentration; Ascorbic acid at 4 µg/mL: reference. A different letter on the table means a significant difference (*p* ≤ 0.05). Results are mean ± SD (*n* = 3).

**Table 4:** % inhibition of Anticancer activity of petals and cores extracts (50 µg/mL) from *P. halepensis*, *P. brutia*, and *P. pinea*.

Extraits	% Inhibition Hela Cell ^a^	% Inhibition HepG2 Cell ^b^	% Inhibition HEK-293 Cell ^c^
PA-P-1SV	76.07 ± 6.81	50.99 ± 5.44	11.01 ± 3.96
PA-C-1SV	67.64 ± 6.32	37.45 ± 4.72	11.05 ± 4.07
PA-P-2SV	75.61 ± 3.88	57.59 ± 0.95	7.22 ± 2.09
PA-C-2SV	50.77 ± 3.43	47.45 ± 3.23	5.27 ± 1.05
PA-P-3SV	21.85 ± 1.51	41.11 ± 4.41	1.43 ± 0.60
PA-C-3SV	21.29 ± 0.95	13.54 ± 4.53	5.34 ± 1.76
PB-P-1SV	69.58 ± 5.25	30.99 ± 5.31	6.78 ± 1.14
PB-C-1SV	65.07 ± 3.98	43.59 ± 3.33	8.99 ± 1.91
PB-P-2SV	60.81 ± 4.44	33.44 ± 2.11	8.04 ± 3.78
PB-C-2SV	56.22 ± 4.72	27.07 ± 3.27	5.05 ± 2.41
PB-P-3SV	19.56 ± 0.78	16.11 ± 2.69	4.26 ± 1.33
PB-C-3SV	22.54 ± 2.59	14.52 ± 3.66	4.83 ± 1.09
PP-P-1SV	74.12 ±5.61	37.51 ± 2.16	5.73 ± 1.11
PP-C-1SV	66.67 ± 5.78	50.08 ± 5.01	2.28 ± 0.76
PP-P-2SV	66.64 ± 3.33	50.83 ± 3.03	9.28 ± 0.71
PP-C-2SV	66.18 ± 3.02	35.18 ± 2.17	6.12 ± 2.19
PP-P-3SV	42.06 ± 5.89	17.98 ± 1.25	2.19 ± 0.48
PP-C-3SV	24.69 ± 1.23	15.57 ± 2.91	3.66 ± 1.17
Tamoxifen	77.72 ± 4.12	70.33 ± 3.91	65.22 ± 2.46

PA: *Pinus halepensis*; PB: *Pinus brutia*; PP: *Pinus pinea*; P: petal; C: core; 1SV: Cyclohexane; 2SV: Ethyl acetate; 3SV: Methanol; Hela cell line: human epithelial cervix carcinoma; HepG2 cell line: hepatic cancer cell line; HEK-293 cell line: human embryonic kidney cell line; Tamoxifen at 100 µg/mL: reference. A different letter on the table means a significant difference (*p* ≤ 0.05). Results are mean ± SD (*n* = 3).

**Table 8:** Correlation coefficients R^2^ of total phenolic content, Antioxidant activity, anticancer activity, and Reducing sugar content from *P. halepensis*, *P. brutia*, and *P. pinea* petals and cores extracts.

Variables	TPC	RSC	% Inhibition DPPH	% Inhibition Hela	% Inhibition HepG2
TPC	1.00	0.92	0.86	−0.84	−0.54
RSC	0.91	1.00	0.83	−0.82	−0.64
% inhibition DPPH	0.86	0.83	1.00	−0.83	−0.74
% inhibition Hela	−0.84	−0.82	−0.82	1.00	0.76
% inhibition HepG2	−0.55	−0.64	−0.74	0.76	1.00

PA: *Pinus halepensis*; PB: *Pinus brutia*; PP: *Pinus pinea*; P: petal; C: core; 1SV: Cyclohexane; 2SV: Ethyl acetate; 3SV: Methanol; TPC: total phenolic content; DPPH: Antioxidant activity; HepG2: anticancer activity; RSC: Reducing sugar content.

## Data Availability

Data will be made available on request.
